# Drink, but don't drive? The alcohol industry’s involvement in global road safety

**DOI:** 10.1093/heapol/czaa097

**Published:** 2020-11-21

**Authors:** Connie Hoe, Niloufer Taber, Sarah Champagne, Abdulgafoor M Bachani

**Affiliations:** Johns Hopkins International Injury Research Unit, Department of International Health, Health Systems Program, Johns Hopkins Bloomberg School of Public Health, 615 N. Wolfe Street, Baltimore, MD 21205, USA

**Keywords:** Alcohol, drink-driving, road safety, industry interference, commercial determinants of health

## Abstract

Drink-driving is a major cause of global road traffic fatalities, yet few countries have laws that meet international best practices. One possible reason is the alcohol industry’s opposition to meaningful policies that are perceived to directly threaten sales. Our primary objectives are to document alcohol industry involvement in global road safety policies and programmes and to critically evaluate the responses of public health and road safety communities to this involvement. Under the guidance of the Policy Dystopia Model, we used a mixed methods approach in which data were gathered from expert interviews and a mapping review of 11 databases, 5 watchdog websites and 7 alcohol industry-sponsored initiatives. Triangulation was used to identify points of convergence among data sources. A total of 20 expert interviews and 94 documents were analysed. Our study showed that the alcohol industry acknowledges that drink-driving is an issue but argues for solutions that would limit impact on sales, akin to the message ‘drink—but do not drive’. Industry actors have been involved in road safety through: (1) coalition coupling and decoupling, (2) information production and management, (3) direct involvement in policymaking and (4) implementation of interventions. Our study also shed light on the lack of cohesion within and among the public health and road safety communities, particularly with regard to the topics of receiving funding from and partnering with the alcohol industry. These results were subsequently used to adapt the Policy Dystopia Model as a conceptual framework that illustrates the ways in which the alcohol industry has been involved in global road safety. Several implications can be drawn from this study, including the urgent need to increase awareness about the involvement of the alcohol industry in road safety and to build a cohesive transnational alcohol control advocacy alliance to curb injuries and deaths related to drink-driving.

**KEY MESSAGES**

- The alcohol industry acknowledges that drink-driving is an issue but argues for solutions that would limit impact on sales. It accomplishes this by being involved in road safety through: (1) coalition coupling and decoupling, (2) information production and management, (3) direct involvement in policymaking and (4) implementation of interventions as well as developing narratives to frame the issue of drink-driving.
- Our study also shed light on the lack of cohesion within and among the public health and road safety communities, particularly with regard to the topics of receiving funding from and partnership with the alcohol industry.
- There is an urgent need to increase awareness about the involvement of the alcohol industry in road safety and build a cohesive transnational alcohol control advocacy alliance to curb injuries and deaths related to drink-driving.

## Introduction

Over the last several decades, global shifts in the profile of harms to health are not only associated with economic development and public health interventions to address diseases but also associated with the increasing consumption of harmful products such as tobacco, alcohol and sugar-sweetened beverages (IHME, 2017). The consumption of these harmful products has been pushed by their commercialization and industrial-level production, backed by industry giants, creating an inherent conflict between profit and public health. These politically and economically influential transnational corporations use an array of tactics and arguments to promote their products to maximize profits (Babor *et al.*, 2015). These tactics and arguments are often successfully leveraged to oppose or weaken the most cost-effective, population- and evidence-based public health policies, as these policies are frequently antithetical to the economic interests of industry (Gilmore *et al.*, 2011; 2015; Esser *et al.*, 2016). Scholars have termed this conflict between public health and private profit the *commercial determinants of health*, defined as ‘strategies and approaches used by the private sector to promote products and choices that are detrimental to health’ (Kickbusch *et al.*, 2016).

The industrial production and wide commercial availability of alcohol present several potential risks to human health. Alcohol serves as a risk factor for over 200 different health conditions across all categories of disease burden, affecting both the incidence and progression of infectious diseases, non-communicable diseases and injuries (Jahiel and Babor, 2007; Rehm *et al.*, 2009; IHME, 2017). Globally, alcohol is implicated in 6% of all deaths, almost 3.3 million deaths, and the loss of over 111 million disability-adjusted life years (IHME, 2017).

Alcohol plays a significant role in road traffic injuries, with between 5% and 35% of all road traffic deaths attributable to alcohol (WHO, 2018b). However, this burden is spread inequitably; while the role of alcohol in road traffic mortality has not changed in high-income countries, alcohol has been playing an increasing role in road traffic injury morbidity and mortality in low- and middle-income countries (LMICs) since 1990 (IHME, 2017).

According to the most recent Global Status Report on Road Safety, only 26% of countries (45 out of 175) have drink-driving policies that align with international best practices (WHO, 2018b). It is also important to note that the most effective and cost-effective interventions for reducing alcohol-related road traffic injuries, such as drink-driving policies, are directed at reducing the availability and sale of alcohol, either overall or to particular vulnerable groups (WHO, 2018a). The success of these population-level, evidence-based public health strategies is a direct threat to the alcohol industry’s interest in maintaining and increasing sales (Casswell *et al.*, 2016). Accordingly, one possible contributing factor to the current state of global drink-driving policy is the alcohol industry’s opposition to meaningful policies that are perceived to directly threaten the industry’s interest (Babor *et al.*, 2015; Esser *et al.*, 2016; Pantani *et al.*, 2017; Babor *et al.*, 2018b). Esser *et al.* (2016), e.g. showed that the majority of alcohol industry’s action to reduce drink-driving globally do not follow evidence-based recommendations.

The primary objectives of this study are to document the involvement of the alcohol industry in global road safety policies and programmes and to critically evaluate the range of collaborative to adversarial responses of the public health and road safety communities to this involvement. It is important to note that the alcohol industry is not a monolithic actor and this study focuses primarily on multinational producers.

Under the guidance of the Policy Dystopia Model, we used a mixed methods approach, including a mapping review and expert interviews to meet the study objectives. Deductive and inductive coding were undertaken, allowing for new themes to emerge. Based on our findings, we adapted the Policy Dystopia Model as a conceptual framework that illustrates the various ways in which the alcohol industry has been involved in global road safety.

### Conceptual model

While, to our knowledge, conceptual frameworks/models specific to alcohol industry involvement in road safety and public health in general do not currently exist, there are conceptual frameworks/models available to analyse corporate political action and interference of other industries (Hillman and Hitt, 1999; Oliver and Holzinger, 2008; Ulucanlar *et al.*, 2016; Liedong *et al.*, 2020). Given the similarities that exist between tobacco and alcohol industry’s approaches to public health, (Bond *et al.*, 2009; Esser *et al.*, 2016; Savell *et al.*, 2016; Hawkins *et al.*, 2018), we elected to use the Policy Dystopia Model as our starting point to guide data collection and analysis (Ulucanlar *et al.*, 2016). Furthermore, while this model is tobacco industry specific, it draws on more general frameworks of corporate political engagement (Hillman and Hitt, 1999).

The Policy Dystopia Model shows that industry crafts narratives that highlight the undesirability of public health policies and conveys them through action-based strategies, including coalition management, information management, direct involvement/influence in policy, litigation and illicit trade (Ulucanlar *et al.*, 2016).

## Methods

Data were gathered from two sources: a mapping review conducted between September and November 2018 and expert interviews carried out from October 2018 to August 2019. The sources were analysed separately and then triangulated.

### Mapping review

A mapping review is used to map out and categorize existing literature on a targeted topic, identifying gaps in knowledge, which serves as a basis for additional research (Grant and Booth, 2009). Based on a review of similar studies, 11 different databases were searched: PubMed/Medline, Scopus, Embase, the Cochrane Library, EconLit, Cinahl, Science Direct, Pro Quest Digital Dissertations, Global Health Observatory, Grey, Literature Report and Communication Initiative Network. The search terms were organized around three constructs—(1**)** alcohol, (2**)** industry and (3**)** traffic. Unpublished studies and other grey literature were also obtained from experts, and a hand search of alcohol watchdog websites, alcohol industry-sponsored initiatives and key articles (Table 1).

**Table 1:** Alcohol watchdog websites and alcohol industry-sponsored initiatives

Alcohol watchdog websites	Alcohol industry-sponsored initiatives
US Alcohol Policy Alliance Alcohol justice International Organisation of Good Templars (IOGT) International Global Alcohol Policy Alliance Institute of Alcohol Studies	Together for Safer Roads Worldwide Brewing Alliance International Alliance for Responsible Drinking Foundation for Advancing Alcohol Responsibility Diageo Pernod Ricard AB InBev Foundation

The inclusion criteria for the mapping review were that the paper was published during the period of 2000–2018 (inclusive) to include literature produced in the years after road safety first became an international concern in 1999 (Walter *et al.*, 1999), in the English language, and examined the involvement of the alcohol industry in road safety policies and programmes. Studies that aimed to understand alcohol industry activities with no link to road safety were excluded. Similarly, studies pertaining to road safety with no link to the alcohol industry were also excluded.

Titles and abstracts were downloaded to Covidence (https://www.covidence.org/home)—a Cochrane technology platform and recommended tool that can be used to help assist in primary screening and data extraction. Duplicates were removed in the process. Two study team members (S.C. and N.T.) screened all citations by title and abstract and removed any articles that did not meet inclusion criteria, and a random check was conducted by a third member of the research team (C.H.). Potentially eligible full-texts were obtained, and eligibility was determined by the study team, with any disagreements resolved through discussions. A ‘hand-searching’ technique was also used, in which we reviewed reference lists of particularly relevant articles to identify additional published literature. Eligible studies were reviewed in full and analysed using deductive coding based on the Policy Dystopia Model and inductive coding.

### Expert interviews

Documents, watchdog resources and industry websites were reviewed to purposively identify experts who have extensive knowledge of the involvement of the alcohol industry in road safety policies and programmes. Snowball sampling was also employed during data collection, such that experts who have been interviewed were asked to assist with identifying other individuals who may be appropriate for the study. Academic experts were contacted directly, whereas many non-governmental organizations (NGOs), Social Aspect/Public Relations Organizations (SAPROs) and alcohol companies were contacted through their organization’s general contact page. All identified individuals were invited to participate in the study via email, phone call or in person.

Semi-structured interviews were conducted with the experts by phone or Skype using a field guide that included questions pertaining to the alcohol industry’s involvement in road safety policies and programmes and responses of the public health and road safety communities. On average, interviews lasted ∼30 min.

Recorded interviews were transcribed, after which transcripts and interviewers’ notes were analysed using inductive coding, as well as deductive coding based on the Policy Dystopia Model. The qualitative analysis computer software HyperRESEARCH was used to facilitate the process.

### Triangulation

Triangulation was subsequently applied to identify points of convergence between the expert interviews and mapping review. Although there was overlap between the experts interviewed and the authors of some of the literature we reviewed, the two sources offered different perspectives and information. The process of triangulation involved summarizing and categorizing the data by the domains of the Policy Dystopia Model, as well as new themes that emerged. The deductive codes ‘information management’ and ‘direct involvement in policymaking’ were maintained. We divided ‘information management’ into ‘information production’ and ‘information management’ as these two types of involvement emerged to be related but distinct. The code ‘coalition coupling and decoupling’ was adapted from the Policy Dystopia Model’s ‘coalition management’ to properly encapsulate the alcohol industry’s involvement in road safety. ‘Illicit trade’ and ‘litigation’ were removed as the alcohol industry was not found to be involved in road safety in those two ways. ‘Implementation of interventions’ and the types of arguments all emerged inductively. Subsequently, a framework specific to this topic was developed based on these findings.

### Ethical review

This study was deemed non-human subjects research by the Institutional Review Board of the authors’ institute. Interviewers went through an oral consent form with the interviewees at the beginning of the study.

## Results

**Figure 1:**
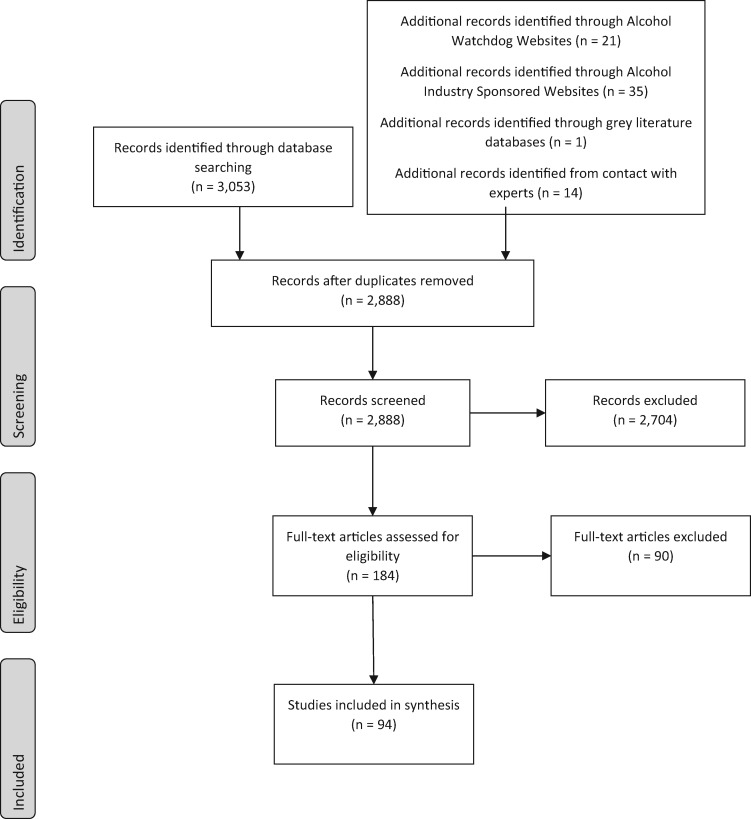
Flow diagram

A total of 94 documents met inclusion criteria and were included in this review; of the 94 documents, 33 were peer-reviewed research articles (Figure 1 and Table 2).

**Table 2:** Characteristics of all reviewed documents (*N* = 94)

	Number of articles	Peer-reviewed research articles
Year published		
2000–2005	9	4
2006–2010	21	15
2011–2018	64	14
Study location		
African Region	7	3
Western Pacific Region	6	4
European Region	9	3
Global/multiple regions	19	4
Latin America/Caribbean region	5	2
USA and Canada	21	9
South East Asian Region	3	0
N/A	24	8
Article type		
Peer-reviewed research	33	33
Editorials	7	Not Applicable
Grey literature	54	Not Applicable
Research design		
Interviews and case studies	Not Applicable	2
Content and document analyses	Not Applicable	11
Cross-sectional	Not Applicable	2
Reviews (e.g. critical, literature, systematic, state-of-the-art review)	Not Applicable	10
Mixed methods	Not Applicable	4
Others (qualitative stakeholder engagement and consensus, econometrics)	Not Applicable	4

The majority of peer-reviewed research articles were published between 2006 and 2018 (*n* = 29) and examine the involvement of alcohol industry on road safety policies and programmes in the USA and Canada (*n* = 9); few focused on LMICs. While a range of study designs have been used, content and document analyses (*n* = 11) and reviews (*n* = 10) were the most common approach (Table 2).

As shown in Table 3, a total of 20 (*n* = 20) interviews were conducted with 7 key informants from NGOs (*n* = 7), 3 from SAPROs/alcohol companies (*n* = 3) and 10 from academia (*n* = 10). Note that two of the academics are also members of alcohol watchdog groups. Fewer industry members and affiliates responded and/or agreed to be interviewed for this project. Correspondingly, non-industry affiliates are more heavily represented (*n* = 12).

**Table 3:** Characteristics of experts interviewed and invited for interview

	Number interviewed (*N* = 20)	Invited for interview (*N* = 53)
Field		
Academia	10	20
NGOs who accept industry funding	5	5
NGOs who do not accept industry funding	2	12
SAPRO/alcohol company	3	16
Expert’s Location		
Global	1	8
North America	9	14
African Region	5	6
European Region	1	4
Western Pacific Region	3	5
South East Asia Region	1	7
South America	0	9

### Involvement of the alcohol industry in global road safety

In this section, we will first describe results pertaining to the various ways in which the alcohol industry has been involved in road safety (Table 4), followed by the different types of arguments used by the industry to frame the issue of drink-driving (Table 5). Subsequently, we present the responses of the public health and road safety communities to this involvement.

**Table 4:** Strategies used by alcohol industry actors to influence road safety policies and programmes

Strategies	Detail
Coalition coupling and decoupling	Alcohol industry actors partner with an array of road safety and public health stakeholders, including international, governmental and NGOs. These partnerships involve joining or forming multi-stakeholder coalitions, providing financial support, giving technical assistance, creating ‘astroturf’ organizations and forming SAPROs (ID 1, 3, 5, 7, 9, 11,15, 19, 21) (Stockwell and Crosbie, 2001; Institute for Alcohol Studies, 2002; Solomon *et al.*, 2004; Worldwide Brewing Alliance, 2008; Anderson *et al.*, 2009; Ogazi and Edison, 2012; Pernod Ricard 2012; 2016; Diageo 2013a,b; 2015a,b; 2018; Esser *et al.*, 2016; IOGT International, 2018; McCambridge *et al.*, 2018; Mialon and McCambridge, 2018 )
Information production and management	Alcohol industry actors are involved in research through recruiting scientists to carry out research, funding research directly or through a third party and conducting research itself; and through engaging in reputation management through CSR activities (ID 2, 3, 4, 5, 6, 7, 8, 9, 11, 16, 17, 18, 19, 20) (Pinsky and Laranjeira 2004; Stockwell and Crosbie, 2001; Diageo 2013a; 2018; Mialon and McCambridge 2018; Robaina *et al.*, 2018)
Direct involvement in policymaking	Industry is also directly involved in the road safety policymaking process at both the global and national levels (ID 1, 2, 3, 4, 6, 7, 9, 11, 16, 18, 19) (Sugarman, 2009; Casswell and Thamarangsi, 2009; EUROCARE, 2014; UNITAR, 2016; Babor *et al.*, 2018; McCambridge *et al.*, 2018; Vital Strategies, 2018)
Implementation of interventions	The alcohol industry funds, supports and rolls out drink-driving interventions, including educational campaigns, which often advocates for ‘responsible’ drinking, ride shares and/or designated driver programmes; many of which are mass media and educational programmes that do not affect alcohol sales and are considered ineffective or have limited effectiveness (ID 1, 2, 3, 4, 5, 6, 8, 9, 11, 21) (Howat *et al.*, 2004; Pantani *et al.*, 2012; Babor *et al.*, 2015; 2018; Esser *et al.*, 2016)

**Table 5:** Arguments used by alcohol industry actors to influence road safety policies and programmes

Arguments	Detail
Personal responsibility	Alcohol misuse is an issue of ‘personal responsibility’ around which each consumer must exercise judgement (ID 3, 5, 6, 8, 19) (Anderson, 2004; Room, 2004; Wolburg, 2005; Misell, 2013; Esser *et al.*, 2016; Smith *et al.*, 2006; Robaina *et al.*, 2018)
Moderate majority	The majority of drinkers are moderate and responsible. Harms are caused by an aberrant minority. This ‘moderate majority’ should not be swept up in policies and interventions which are poorly targeted (ID 5, 20) (Misell, 2013)
Legitimate stakeholder	The alcohol industry is a ‘legitimate stakeholder’ in developing alcohol policy (ID 5, 20) (Perl and Brotzman, 2018; Robaina *et al.*, 2018)
(False) choice	There is a ‘(false) choice’ between only two, mutually exclusive options: neo-prohibition and industry self-regulation (Toomey, 2005)
Issue decoupling	‘Issue decoupling’: alcohol misuse is a risk factor for drink driving-related injuries and deaths, but alcohol use is otherwise not problematic (Anderson, 2004; Lindsay *et al.*, 2008; Reiling and Nusbaumer, 2007)
Highlighting other issues	‘Highlighting other issues’: major issues in road safety include drowsy or drug driving, which can be as dangerous as drunk driving (ID 20) (Ogazi and Edison, 2012; AB InBev, 2016; Foundation for Advancing Alcohol Responsibility, 2018)

#### Coalition coupling and decoupling

The alcohol industry partners with an array of road safety and public health stakeholders including international (IOGT International, 2018), governmental [identification (ID) 7, 9, 11,15, 19, 21] (Worldwide Brewing Alliance, 2008; Pernod Ricard, 2012; Diageo, 2013a,b; 2015a,b; 2016; 2018) and NGOs (ID 5, 15, 19) (Diageo, 2013b). Partnering with these groups involves providing funding (ID 7, 15, 19) and support to carry out road safety interventions (ID 11, 21) and to pass legislations (ID 15). The industry also participates in road safety coalitions (ID 3, 15) or creates them where they do not exist (ID 15), bringing in influential organizations of transnational governance like the World Bank (ID 19). According to some key informants, the motivations behind these partnerships are for the industry to garner legitimacy (ID 19), present themselves as equal partners (ID 5) and market themselves as good corporate citizens (ID 3).

*We felt that a strategic partnership with them [government entity] would actually elevate the work that we are doing, but also give more credibility and integrity to the work that we are doing* (SAPRO/Alcohol Company).

An industry-affiliated key informant, on the other hand, explained that a motivation is to address the harms caused by alcohol when the product is used in a harmful way:

*Well, my view is that the alcohol industry like any other industry sector, has to be good corporate citizens and if the consumption of their products, gets utilized in a harmful way leads to social ills and challenges, then, they have to make some investment in trying to redress that* (SAPRO/Alcohol Company).

According to another key informant, the industry also engages in coalition decoupling by providing financial support and technical assistance to specific government sectors (e.g. Transportation, Law Enforcement, Ministry of Trade), such that when the Ministry of Health, e.g. proposes an alcohol control related legislation, these industry-supported sectors will advocate for watering down the law (ID 11).

*[When] alcohol is being regulated effectively, then you will see the same government [sector] coming in to suppress the Ministry of Health to relax the regulation because the alcohol industry is supporting another arm of government* (Non-Industry-Affiliated NGO).

Other ways in which the alcohol industry engages in coalition management is through setting up SAPROs (ID 19) (Anderson *et al.*, 2009; Ogazi and Edison, 2012; Esser *et al.*, 2016; McCambridge *et al.*, 2018; Mialon and McCambridge, 2018) and being actively involved in so-called ‘astroturf’ organizations posing as trade groups such as the American Beverage Institute (ABI) (ID 1) or the Australian Associated Brewers (Stockwell and Crosbie, 2001; Solomon *et al.*, 2004). These groups represent the industry during policy debates and in public and social discourse (Institute for Alcohol Studies, 2002). A key informant described ABI as the industry’s ‘junk yard dog’ (ID 1) as ABI is paid to oppose effective alcohol control laws that will affect sales (ID 1).

#### Information production and management

The industry is also involved in road safety through information production; this entails being involved in research (Stockwell and Crosbie, 2001; Mialon and McCambridge, 2018) through recruiting scientists to carry out research (ID 2, 4, 5, 6, 8, 18), funding research directly or through a third party (ID 3) and conducting research itself (ID 3, 4, 16, 20). One key informant noted that having funds channeled through a third party is strategic on the part of the alcohol industry, as being one step removed helps create legitimacy through at least the appearance of independence, which in turn makes the funding more acceptable to NGOs and governments (ID 3). Non-industry-affiliated public health key informants also explained that these information production-related activities are used to protect and market the industry, bring academic authority to industry-related findings and undermine public health research, which focuses on evidence-based, population-wide interventions (ID 2, 3, 4, 5).

*They’re conducting research and calling it research but they’re using it for marketing purposes* (Public Health Academic).

The alcohol industry also manages its reputation as a good corporate citizen through engaging in corporate social responsibility (CSR) activities (Mialon and McCambridge, 2018; Robaina *et al.*, 2018). Key informants noted that drinking and driving is an issue that the alcohol industry cannot outright deny (ID 8, 9). Accordingly, it has become an area where they like to draw the public’s attention to in terms of alcohol harms. It is an area the industry is investing in to ‘make them look good’ (ID 3). Examples include donating breathalyzers to police departments (ID 7, 19) (Pinsky and Laranjeira, 2004; Diageo, 2013a; 2018), putting money into the road safety sector (ID 8), providing training and technical assistance to government (ID 11), sponsoring campaigns (ID 4, 6) and promoting activities that have high public relations visibility (ID 3).

#### Direct involvement in policymaking

The alcohol industry is also directly involved in the road safety policymaking process at both the global and national levels. With regard to the former, there is evidence to suggest that the alcohol industry is very active at the United Nations level (UNITAR, 2016; Vital Strategies, 2018). Moreover, Jean Todt, the special envoy for road safety, is the president of the Federation Internationale de l’Automobile (FIA). FIA licences and sanctions Formula One, which receives sponsorship from Heineken (EUROCARE, 2014).

At the national level, alcohol industry representatives and lobbyists also oppose or attempt to water down population-level policies that may reduce profits (ID 1, 3, 9, 16) (Casswell and Thamarangsi, 2009; Sugarman, 2009; McCambridge *et al.*, 2018). Findings showed that several mechanisms have been used to achieve these policy objectives. Industry representatives, lobbyists and other affiliates engage and cultivate positive relationships with decision-makers, setting up forums, events and/or coalitions to increase interactions among them (ID 2, 4, 11, 18, 19). Technical assistance and other types of incentives, including donations to political campaigns, meals, cars, travel and entertainment, have been offered to these ‘duty bearers’ (ID 1, 3, 4, 6, 7, 11, 18) (Babor *et al.*, 2018b).

*They [alcohol industry] wine and dine state legislators and they contribute to their campaign* (Public Health Academic).

A ‘revolving door’ between government and industry was also identified by one of the key informants as a mechanism used; this describes the overwhelming majority of industry lobbyists in 2016, who were government officials who left public office and were subsequently employed by the alcohol industry (ID 4) (Babor *et al.*, 2018b).

#### Implementation of interventions

The alcohol industry funds, supports and rolls out drink-driving interventions, including educational campaigns, which often advocates for ‘responsible’ drinking, ride shares and/or designated driver programmes (Howat *et al.*, 2004; Pantani *et al.*, 2012; Babor *et al.*, 2015; 2018a). These activities are, at times, carried out with key road safety stakeholders (ID 11, 21). According to non-industry-affiliated public health key informants, while there are a few groups that focus primarily on targeted evidence-based interventions, most of the interventions supported by the alcohol industry are mass media and educational programmes that do not affect alcohol sales and are considered ineffective, or have limited or unknown effectiveness, while allowing the industry to maintain its reputation as a good corporate citizen (ID 1, 2, 3, 4, 5, 6, 8, 9).

*Strategies that the alcohol industry uses are mostly educational programs, to me they are really programs that draw attention away from changing the availability of alcohol, changing the supply of alcohol, changing access to alcohol in the community. I really think of them as distraction techniques* (Public Health Academi*c*).

Esser *et al.* (2016) also found, after analysing the content of 266 alcohol industry-sponsored global initiatives to reduce drink-driving, that the majority of these initiatives are inconsistent with evidence-based public health recommendations; only 0.08 (*n* = 2) were.

## Arguments

The alcohol industry also develops arguments to frame the issue of drink-driving. The identified arguments are as follows: (1**)** alcohol misuse is an issue of *personal responsibility* (ID 3, 5, 6, 8, 19) (Anderson, 2004; Room, 2004; Wolburg, 2005; Misell, 2013; Esser *et al.*, 2016; Smith *et al.*, 2006; Robaina *et al.*, 2018); (2**)** the majority of drinkers are moderate and responsible (*moderate majority*) (ID 5, 20) (Misell, 2013); (3**)** the alcohol industry is a *legitimate stakeholder* (ID 5, 20) (Perl and Brotzman, 2018; Robaina *et al.*, 2018); (4**)** there is *a* (*false*) *choice* between neo-prohibition and industry self-regulation (Toomey, 2005); (5**)** alcohol misuse is primarily a risk factor for drink-driving-related injuries and deaths, rather than for other health diseases (*issue decoupling*) (Anderson, 2004; Reiling and Nusbaumer, 2007; Lindsay *et al.*, 2008); and (6**)** major issues in road safety include drowsy or drugged driving or alcohol use and walking (*highlighting other issues*) (ID 20) ( Ogazi and Edison, 2012; AB InBev, 2016; Foundation for Advancing Alcohol Responsibility, 2018).

### Responses from the public health and road safety communities

The public health and road safety communities have been rather fragmented in their responses to the involvement of the alcohol industry in road safety, particularly as it relates to funding and partnership.

As highlighted by some key informants, the main advantage of partnering with the alcohol industry is funding (ID 12; 16; 20; 21) as there are few funders in the area of road safety, particularly in LMICs (ID 16; 21). Of the informants who do receive alcohol funding, several highlighted that they were able to maintain independence and were able to retain industry funding even when advocating for population-wide and evidence-based interventions such as taxation and advertising restrictions (ID 15, 16, 17).

*I believe we have made great use of the funding that came from the industry [* **_*…*_** *] [and] I wouldn’t have been able to promote and to push for [drink driving legislations] if I had not had this funding* (Industry-Affiliated NGO).

*They [the alcohol industry] had their opinion and their view and they did their lobbying and I had my opinion and my view and I did my advocating* (Industry-Affiliated NGO).

One key informant noted that despite being able to maintain independence, his/her organization cut ties with the alcohol industry due to pressure from the international public health and road safety communities (ID 16).

On the other hand, non-industry-affiliated public health key informants underscored the inherent conflict of interest between public health and alcohol industry (ID 19, 20), highlighting the range of public health issues resulting from alcohol use. They argued that accepting funding from the industry will necessarily make recipients more susceptible to industry influence, whether they are aware of such influence (ID 3, 18, 20).

*If we were taking money from the alcohol industry and if they came to us and said:* hey we want you to cool it on the ignition interlocks*, you know and if we had built a sizeable part of our revenue around that [funding source], it would be very difficult to say no* (Non-Industry-Affiliated NGO).

With regard to partnership, some key informants argued, given that road safety is a multifaceted issue requiring the input of many stakeholders (ID 13), it is essential that private sector support can elevate the confidence of government officials (ID 13). Non-industry-affiliated public health key informants, on the other hand, explained that partnership helps legitimize the industry’s position as a stakeholder in the eyes of other members of civil society or government (ID 19), as well as in the eyes of potential consumers, effectively becoming an opportunity for marketing (ID 11).

**Figure 2:**
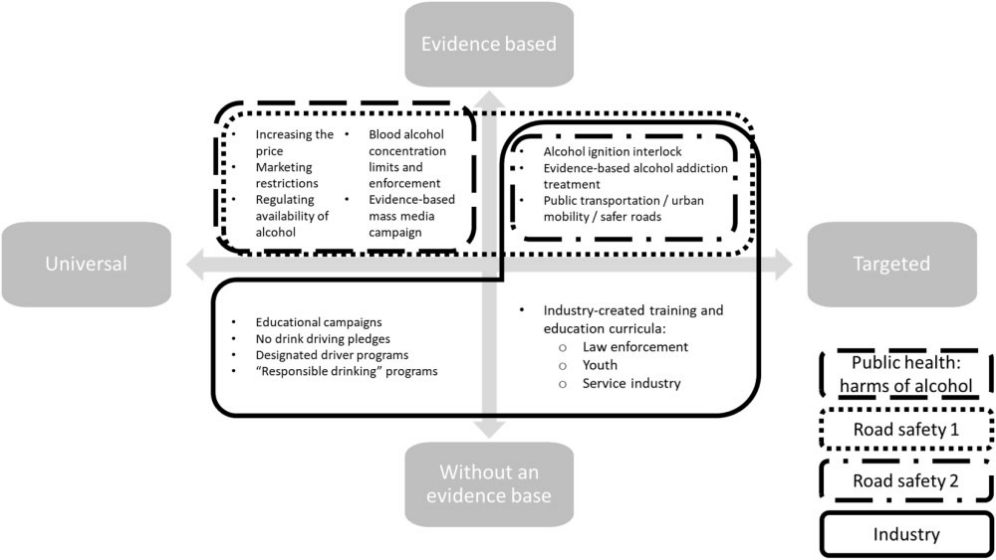
Types of coalitions involved in road safety

Fragmentation was also apparent between the public health and road safety communities. Some groups within the road safety community decouple the issue from other alcohol problems (ID 9, 15, 17, 19), arguing that it goes beyond the organization’s mandate (ID 17) and that there are more proximate steps to tackling road safety than focusing on population-level interventions (ID 15). Accordingly, as illustrated in Figure 2, there appears to be four distinct coalitions (public health, road safety 1, road safety 2 and industry), each with their own sets of problems, definitions and solutions. The public health coalition, e.g. focuses on the harms of alcohol in general and appears to support universal/population-level, evidence-based interventions. The road safety 1 coalition overlaps with the public health coalition in support of universal, evidence-based interventions as well as targeted evidence-based interventions. Road safety 2 coalition, on the other hand, focuses on targeted evidence-based interventions and tends to be organizations working specifically on road safety and not public health in general. Lastly, the industry coalition appears to support targeted evidence-based interventions and universal and targeted interventions without an evidence base.

## Discussion

**Figure 3:**
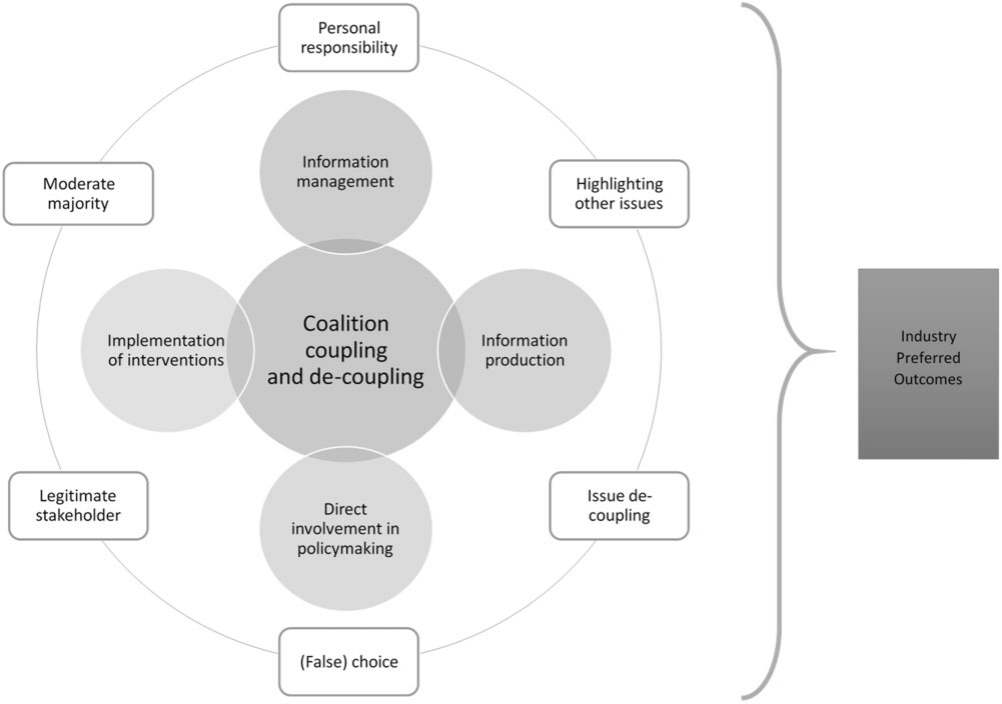
Framework for alcohol industry’s involvement in road safety

The alcohol industry has been involved in global road safety through: (1**)** coalition coupling and decoupling, (2**)** information production and management, (3**)** direct involvement in policymaking and (4**)** implementation of interventions, supported by their arguments (Figure 3). These findings are consistent with the strategies identified by Babor *et al.* (2018) and akin to the involvement of the alcohol industry in public health in general (McCambridge *et al.*, 2018; McCambridge and Mialon, 2018; Mialon and McCambridge, 2018). Our mapping review also revealed several gaps in literature. There were only 33 peer-reviewed research articles on this topic and the majority focused on the North American region despite the fact that LMICs have higher rates of alcohol attributable mortality (Rehm *et al.*, 2009), possess weak governance structures and are sites where transnational alcohol corporations are increasingly turning their attention towards (Babor *et al.*, 2010; 2015). Moreover, few policy analyses exist, which limits our understanding of the road safety policy processes and the specific ways in which the alcohol industry, e.g. coordinates and implements its strategies through proxy organizations.

While the Policy Dystopia Model was a useful starting point and our findings are generally consistent, there were several differences (Figure 3). The Policy Dystopia Model posits that the tobacco industry constructs a dystopian narrative that highlights the dysfunctional future that will arise if tobacco control policies were pursued and convey them through action-based strategies to diminish or eliminate policy impact on business. While we found that the alcohol industry also develops narratives and conveys them through some similar mechanisms (e.g. coalition management, information management, direct involvement and influence in policy), the alcohol industry did not have to fully develop an overarching narrative of a dysfunctional future around the issue of road safety as there was more room to shift the debate towards harm reduction. Instead for road safety, the alcohol industry admits that drink driving is an issue and focuses its arguments on solutions that will ultimately lessen or eliminate impact on sales, akin to the message ‘drink, but do not drive’. Our findings show that these narratives are consistent with the three strands of arguments—(1**)** positioning the alcohol industry as a key stakeholder, (2**)** downplaying the scale and scope of the alcohol problem and (3**)** focusing on personal responsibility rather than a population-level approach—uncovered by McCambridge *et al.* (2018) when exploring the alcohol industry involvement in policymaking in general. It is important to note that the rhetoric of personal responsibility is also one that has been heavily utilized by other industries including tobacco (Mejia *et al.*, 2014; Friedman *et al.*, 2015) and food (Brownell *et al.*, 2010) and linked with conservative ideology (Mejia *et al.*, 2014; Ulucanlar *et al.*, 2016).

Our study also showed that coalition coupling and decoupling appeared to the enabler for all other types of involvement. There also appeared to be a heavy emphasis on the implementation of interventions. These differences could be due to the fact that tobacco control has a Framework Convention and alcohol control does not; this global treaty includes Article 5.3, which obligates parties of the treaty to protect tobacco control policies from industry interference by, e.g. rejecting partnerships with the industry (World Health Organization, 2008). Moreover, the Policy Dystopia Model focuses on tobacco control policies more broadly and this study explores how the alcohol industry has been involved in one policy area (road safety), which, according to our finding, the industry has tried to decouple from other public health issues. Likewise, another hypothesis is that the alcohol industry can steer the focus of the drink-driving problem on to the performance of a specific activity—driving—after substance consumption, instead of on the problem of consumption.

Given these differences, we modified both the visual and content of the Policy Dystopia Model to more aptly represent the alcohol industry’s involvement in road safety (Figure 3). In Figure 3, we highlighted the six key narratives that emerged from our study. Unlike the original Policy Dystopia Model, which used arrows to link arguments to specific model components, we displayed these narratives as an outer ring, illustrating that these arguments are conveyed through all five ways in which the alcohol industry has been involved in road safety. We also emphasized the interconnected and mutually reinforcing nature of all the action-based strategies, and the centrality of coalition coupling and decoupling as an enabler for all other strategies, by placing coalition management at the centre of several overlapping circles.

Our study also shed light on the lack of cohesion within and among the public health and road safety communities, particularly with regard to the topics of receiving funding from and partnership with the alcohol industry. Interestingly, this fragmentation is also reflected in the larger alcohol control and road safety movements. When comparing the global alcohol and tobacco control efforts, Gneiting and Schmitz (2016) found that unlike the case of tobacco control, alcohol control has struggled to garner consensus and ‘today, three distinct approaches to alcohol harm – public health, individual (moral responsibility) and medical treatment – are advanced by separate groups’ (p. i102).

There are several limitations associated with the study. First, this study focused on large multinational producers and industry engagement with road safety likely differs depending on size, international reach, market share and product. Second, many potential industry and industry-affiliated key informants and LMIC civil society actors did not respond to interview requests or declined participation; as such their perspectives might not have been fully captured. Third, the limitations of our review reflect the limitations of the existing literature. As it is challenging to observe ‘behind the scenes’ industry involvement and there are no internal company documents akin to those available for tobacco companies, the existing studies only captured data that are relatively accessible. As such, the types of involvement included in this report may not be exhaustive. Furthermore, we did not assess the quality of the existing literature. Future studies can be undertaken to address this gap. Finally, only English documents were reviewed, which might have resulted in loss of some details.

## Conclusions

This study shed light on the involvement of the alcohol industry in global road safety policies and programmes. Based on our findings, we modified the Policy Dystopia Model to reflect this topic. Alcohol control advocates can use the adapted framework to identify key entry points and devise targeted strategies. Future research can explore the applicability of this framework to their context.

This study also uncovered the range of responses from the public health and road safety communities regarding this involvement. Political scientists have long argued that cohesiveness within the policy community is a key factor for political prioritization, without which meaningful policy change may not take place (Sabatier, 1988; Shiffman and Smith, 2007; Kingdon, 2011). From this perspective, our findings suggest that public health and road safety communities need to generate consensus and rally in one voice; this includes the formation of a cohesive transnational alcohol control advocacy network.

Another recommendation would be for public health-focused advocates to ‘call in’ (an attempt to motivate outlying organizations to join in on one’s approach to the alcohol industry) rather than ‘call out’ (point out the flaws or shortcomings of another group’s approach) (Ahmad, 2015; Ross, 2019) those in the road safety community and groups focused on targeted interventions (Figure 2). This could be accomplished through a stronger focus on building advocacy alliances and on information dissemination emphasizing the importance of population-based and targeted evidence-based practices, as well as the issues associated with the ‘drink but do not drive’ narrative.

Moreover, given that our mapping review has identified several gaps in literature, more studies in LMICs are needed. Future studies that use a policy analysis approach will also be critical as they will help unveil the actors, processes and determinants that facilitate evidence-based policy change and implementation (Gilson and Raphaely, 2008) despite opposition.
